# Current status of Zika vaccine development: Zika vaccines advance into clinical evaluation

**DOI:** 10.1038/s41541-018-0061-9

**Published:** 2018-06-11

**Authors:** Alan D. T. Barrett

**Affiliations:** Sealy Institute for Vaccine Sciences and Department of Pathology, University of Texas Medical Branch, Galveston, TX 77555-0436 USA

## Abstract

Zika virus (ZIKV), a mosquito-borne flavivirus, was first identified in the 1940s in Uganda in Africa and emerged in the Americas in Brazil in May 2015. In the 30 months since ZIKV emerged as a major public health problem, spectacular progress has been made with vaccine development cumulating with the publication of three reports of phase 1 clinical trials in the 4th quarter of 2017. Clinical trials involving candidate DNA and purified inactivated virus vaccines showed all were safe and well-tolerated in the small number of volunteers and all induced neutralizing antibodies, although these varied by vaccine candidate and dosing regimen. These results suggest that a Zika vaccine can be developed and that phase 2 clinical trials are warranted. However, it is difficult to compare the results from the different phase 1 studies or with neutralizing antibodies induced by licensed flavivirus vaccines (Japanese encephalitis, tick-borne encephalitis, and yellow fever) as neutralizing antibody assays vary and, unfortunately, there are no standards for Zika virus neutralizing antibodies. In addition to clinical studies, substantial progress continues to be made in nonclinical development, particularly in terms of the ability of candidate vaccines to protect reproductive tissues, and the potential use of monoclonal antibodies for passive prophylaxis.

## Introduction

Spectacular progress has been made in the development of a Zika vaccine over the past 30 months. Vaccine development started in the second half of 2015 with the determination of the genomic sequences of Zika virus (ZIKV) isolates in Brazil and cloning of ZIKV genes in to a variety of vectors and the first reports of phase 1 clinical evaluation of vaccine candidates have been published in the 4th quarter of 2017. Over 45 candidates have been evaluated in the discovery phase, over 25 have been in nonclinical development, and at least nine are in clinical evaluation (see Table 1 for an overview of those in clinical evaluation). Nonclinical studies supporting advancing to clinical trials include interferon receptor deficient immunocompromised mice and non-human primates (NHPs) showing that a candidate vaccine will result in undetectable viremia following challenge with a wild-type strain of ZIKV. In addition, the candidate vaccine will induce a reasonable titer of neutralizing antibodies that would be indicative of protective immunity and sera from vaccinated animals will passively protect animals of the same species from ZIKV challenge as defined by undetectable viremia (see ref. ^1^ for a summary).

**Table 1:** Summary of Zika vaccines in clinical trials

Vaccine	Adjuvant	Developer	Regimen (weeks/route)	Phase	Clinical trial	Publication
GLS-5700 Synthetic ZIKV prM/E	None	Inovio and Gene One Life Sciences	1, 2 mg/ID 2 mg/ID	1	NCT02809443 NCT02887482	Ref. ^3^
VRC-ZKADNA085-00-VP	None	NIH/VRC	0 + 8, 0 + 12, 0 + 4 + 8, 0 + 4 + 20 4 mg/IM/both arms	1	NCT02840487	Ref. ^5^
VRC-ZKADNA090 -00-VP ZIKV wt prM/E	None	NIH/VRC	0 + 4 + 8 4 mg/IM Needle vs. PharmaJet	1	NCT02996461	Ref. ^5^
VRC-ZKADNA090-00-VP ZIKV wt prM/E	None	NIH/VRC	0 + 4 + 8 4 mg vs. 8 mg/ IM/Both arms PharmaJet	2	NCT03110770	
Inactivated ZIKV (MR8766)	Alum	Bharat Biotech	0 + 30 days 2.5 vs. 5 vs. 10 µg	1	CTRI/2017/05/008539	
Inactivated ZIKV (VLA 1601)	Alum	Valneva (+Emergent Biosolutions)	0 + 1 vs. 0 + 4 3 vs. 6 antigen units	1	NCT 03425149	
Zika purified inactivated vaccine [ZPIV]	Alum	WRAIR	0, 0 + 1, 0 + 2, 0 + 4 5 µg/IM 0 + 4 2.5 vs. 5 vs. 10 mg/IM 0 + 4 5 µg/IM (Puerto Rico) 0 + 4 5 µg/IM (±YF Vax; ±Ixario) ZPIV booster at 1 year	1	NCT02937233 NCT02952833 NCT03008122 NCT02963909	Ref. ^4^
Inactivated ZIKV (TAK-426)	Alum	Takeda	0 + 4 2 vs. 5 vs. 10 µg/IM	1	NCT03343626	
Live Measles virus-Zika [MV-Zika]	None	Themis	Low vs. high dose 0 vs. 0 + 4	1/2	NCT02996890	
mRNA	None	Valera/Moderna	mRNA-1325	1/2	NCT03014089	

*ID* intradermal, *IM* intramuscular

In July 2016 the World Health Organization (WHO) published a Target Product Profile (TPP) that presented both the minimal and preferred characteristics of a Zika vaccine that would be targeted to protect against congenital Zika syndrome in an emergency scenario. In early 2017, the WHO published an updated TPP for a Zika vaccine to be used in an outbreak response scenario.^2^ Briefly, the indication for use would be to prevent clinical illness in individuals aged 9 and over in 80% of the population (or minimum of 70%) based on the assumption that a reduction in viremia to a particular level (undetectable?) would be associated with prevention of clinical illness and infection of the fetus for at least one year post the primary vaccine series. Based on licensed flavivirus vaccines (Japanese encephalitis [JE], tick-borne encephalitis [TBE] and yellow fever [YF]), it is expected that neutralizing antibodies will be a surrogate of (protective) immunity.

## Clinical studies

A number of the candidate vaccines have advanced in to clinical evaluation (summarized in Table 1). Briefly, four types of vaccines have been in phase 1 clinical trials: three DNA, one modified RNA, four purified formalin inactivated virus [PIV], and one live measles-vectored. These have been evaluated in Zika naïve populations, and are subsequently being evaluated in areas where there is flavivirus activity, notably Puerto Rico. In addition, one of the PIVs is being evaluated in subjects who have previously received JE (Ixiaro™ - inactivated) or YF (YF Vax™ - live attenuated) vaccines. In the fourth quarter of 2016, three papers reported the results of phase 1 clinical trials for the DNA and PIV vaccine candidates.^3–5^

Tebas et al.^3^ published results of a phase 1 clinical trial of their prM + E consensus DNA vaccine (GLS-5700)(NCT02809443)(see Table 1) and builds on previously published nonclinical data.^6^ Briefly, Zika naïve subjects received three doses (0, 4, and 12 weeks) of either 1 or 2 mg GS-5700 by the intradermal route. Two weeks post-vaccine dose 3, 62% of subjects had neutralization titers (by microneutralization assay on Vero cells) with titers ranging from 18 to 317; however, an alternative neutralization assay utilizing glioblastoma cells showed that 95% of subjects had neutralization titers >25. Passive protection of mice with sera from 16 subjects showed that all sera protected at least 60% of mice and overall 92% of mice were protected from wild-type ZIKV strain PR209 challenge.

Modjarrad et al.^4^ reported results of three phase 1 clinical trials of a PIV with alum as adjuvant based on a Puerto Rican strain of ZIKV (NCT02937233, NCT02952833, and NCT02963909 (see Table 1). Again, this study builds on published nonclinical data.^7,8^ The Walter Reed Army Institute for Research group have successfully used this approach to develop a PIV for JE that was licensed to Valneva and marketed as Ixiaro™, which is used in a two-dose regimen consisting of 6 µg virus plus 250 µg aluminium hydroxide adjuvant given by the intramuscular route. The current Zika PIV is a similar two-dose regimen, four weeks apart, with 5 µg virus and 500 µg aluminium hydroxide per dose. Ninety-two percent of subjects showed seroconversion measured as neutralization titers >10, 77% showing neutralization titers >100, and GMTs of 173 at 4 weeks post dose two. Neutralization was measured using a microneutralization assay on Vero cells and titers were similar to those induced by Ixiaro™. Passive transfer studies in mice showed that purified IgG from subjects with a neutralization titer of 60 was protective against challenge with 100pfu of strain SPH2015. This titer is higher than the seroprotective titer for JE, TBE and YF vaccines.

Guadinski et al.^5^ reported two phase 1 clinical trials comparing two prM+E DNA vaccine constructs (NCT02996461 and NCT02840487)(see Table 1). VRC 5288 consisted of ZIKV prM+E (French Polynesian strain) with the carboxy-terminal 98 amino acids from JE virus that encodes the stem-anchor region while VRC 5283 consisted entirely of ZIKV prM+E sequence, and was based on successful nonclinical studies.^9^ This multifaceted study also compared different administration regimens (0, 8; 0, 12; 0, 4, 8; 0, 4, 20 weeks, and needle/syringe vs. needle-free). The best results were obtained with VRC 5283 given as a three-dose regimen (0, 4, and 8 weeks) and administered via needle-free injection in both deltoids (2 mg/deltoid) where the vaccine induced neutralizing antibodies and T cell responses in all 14 subjects in this group. The mean neutralization titer was 304 by a GFP reporter assay on Raji cells expressing DC-SIGNR.

The results of these three studies give confidence that an immunogenic Zika vaccine can be developed, however, whether or not an efficacious vaccine can be developed remains to be seen. All three vaccine candidates were safe and well tolerated, and all induced neutralizing antibodies to varying extents depending on the specific candidate and vaccination regimen. Since neutralizing antibodies are the accepted surrogate of (protective) immunity for licensed JE, TBE, and YF vaccines, it is assumed the same will be true for a Zika vaccine. Although this is not known for humans, neutralizing antibodies appear to be the surrogate of protection in nonclinical studies (see ref. ^1^ for a review). As stated above, the Zika PIV gave very similar immunogenicity results to that seen with JE PIV vaccine Ixiaro™, suggesting that inactivated JE and Zika viruses have similar immunogenicity with aluminum hydroxide adjuvant. However, Ixiaro™ has a seroprotective neutralization titer of 10 while it appears that higher levels of neutralizing antibodies (>60) are required to protect against ZIKV infection with all three vaccine candidates in animal models. The increased level of neutralizing antibodies needed for protection in animals may be in part due to the complex pathogenesis of ZIKV, including congenital infections, whereas JE, TBE and YF each have one major tissue tropism only in humans. However, interpretation needs to be made with care as the vaccine developers are using different neutralization assays, which makes any comparisons between candidate Zika vaccines and/or Zika with other flaviviruses very difficult. Much has been written about the process of developing a Zika vaccine (see ref. ^10,11^ for reviews) and while it is clear much progress has been made, undertaking phase 2 clinical trials involving expanded safety and immunogenicity could be undertaken, clinical endpoint studies with an efficacy endpoint would be a difficult task since there has been very little Zika clinical disease after 2016 (http://www.paho.org/hq/index.php?option=com_content&view=article&id=11599&Itemid=41691&lang=en), and is consistent with the periodic activity of some mosquito-borne viruses, such as West Nile virus. This raises the question how will vaccine efficacy be measured? Will an immunological correlate suffice, or will animal studies be used (e.g., U.S. FDA Animal Rule), or will traditional human efficacy studies be required? Given the complex pathogenesis of Zika virus infection, it is difficult to see how human efficacy studies will not be required by National Regulatory Authorities. One approach would be to utilize a human challenge model as has been undertaken for dengue; however, there are ethical issues that need careful consideration. As with dengue, one possibility may be to consider use of one of the candidate live attenuated vaccine strains, but to date these have not been evaluated in humans in phase 1 clinical trials.

## Nonclinical studies

In addition to the publication of clinical data, there have been some important publications on nonclinical results of vaccine candidates. A live attenuated vaccine based on a 10-nucletide deletion in the 3′ untranslated region of the Cambodia FSS13025 strain ZIKV infectious clone (10-del ZIKV) has been developed. Administration of 10,000 ffu by the subcutaneous route induced sterilizing immunity (defined as lack of an anamnestic antibody response following challenge of mice with wild-type ZIKV) in a mouse model and a very high titer of neutralizing antibodies (>5000 by a mCherry reporter assay), plus the virus was not mosquito competent.^12^ A subsequent study showed that administration of 1000 ffu of this vaccine candidate in rhesus macaques by the subcutaneous route resulted in a low viremia in only 1 of 4 NHPs; however, neutralization titers were low (100 by the mCherry reporter assay) in vaccinated animals. The NHPs were challenged with wild-type ZIKV on day 56 post-immunization and no viremia (vRNA) was detected but vaccinated animals showed an anamnestic neutralizing antibody response following wild-type ZIKV challenge indicating there was not sterilizing immunity and there was ZIKV replication in animals.^13^ Interpretation of the results in NHPs is difficult as the vaccine dose in NHPs was 10-fold less than that used in mice.

A novel approach to vaccines has been developed in the last few years based on the utilization of viral RNA of RNA viruses as a vaccine. In the case of Zika, two candidate modified RNA vaccines have been reported.^14,15^ The vaccine generated by Richner et al.^14^ consists of a lipid-encapsulated nanoparticle (mRNA LNP) given by the intramuscular route containing modified mRNA encoding the prM/E genes that are expressed in cells to produce virus-like particles.^14^ The modified mRNA is non self-amplifying and has an optimized open reading frame that encodes prM/E plus 5′ and 3′ untranslated regions that optimize translation and intracellular stability, and proprietary nucleoside modification to prevent induction of an innate immune response. Two doses (0, 3 weeks) of 2 or 10 µg of the mRNA LNP vaccine in mice induced high titers of neutralizing antibodies in mice (10,000 by PRNT_50_ on Vero cells) and sterilizing immunity on wild-type ZIKV challenge. The second mRNA vaccine was generated by Pardi et al.^15^ and also consists of a mRNA LNP but the ZIKV RNA is based on a French Polynesian isolate from 2013 and contains the modified nucleoside 1-methylpseudouridine, which does not induce the innate immune response and increases mRNA translation. The Pardi et al. candidate vaccine was evaluated in mice and NHPs. One dose of 30 µg of mRNA LNP given by the intradermal route induced neutralization titers in mice of 1100–1300 by standard PRNT_50_. NHPs were immunized with either 50, 200, or 600 µg mRNA LNP by the intradermal route and induced neutralization titers around 400 and most animals has undetectable viremia on wild-type ZIKV challenge.

To date, the 10-del ZIKV^12^ and Richner et al.^14^ mRNA LNP vaccines are the only vaccine candidates to induce sterilizing immunity as determined by lack of anamnestic neutralizing antibody response upon ZIKV challenge, but only in mice, whereas other vaccine candidates do not demonstrate anamnestic neutralizing antibody responses upon challenge indicating the challenge virus was able to initiate infection. It is notable that these candidate vaccines induced very high levels of neutralizing antibodies in the mouse model, which may contribute to the results obtained.

An important question that needs to be addressed is durability of immunity induced by the vaccine candidates as studies to date have looked at immunity for a relatively short period of time after the last dose of vaccine. Recently, Abbink et al.^16^ followed up their earlier study in NHPs^8^ to compare immunity induced by candidate DNA, PIV and rhesus adenovirus Zika vaccines at one-year post immunization of NHPs. Interestingly, the two dose PIV and one dose rhesus adenovirus vectored vaccines still elicited protection while the two-dose regimen of the DNA vaccine resulted in waning antibody levels that were not protective over time. Using a microneutralization assay on Vero cells, the authors found that a neutralization titer around 100 was needed to protect NHPs at one-year post immunization, which is consistent with their earlier study.^8^ It should be emphasized that the DNA vaccine used here is not the same as either used in the clinical trials and so the translation of the results of the Abbink et al. DNA vaccine to other DNA vaccines is not known.

A Zika vaccine that protects against Congenital Zika Syndrome will have to prevent infection of reproductive tissues and so studies have been published with DNA, 10-del ZIKV, NS1 ZIKV (a live attenuated vaccine based on elimination of the two glycosylation sites in nonstructural protein 1 [NS1] of the Cambodia FSS13025 strain ZIKV infectious clone) and mRNA LNP candidate vaccines in mouse pregnancy and testes models.^17,18^ These studies show that the candidate vaccines prevent pregnancy transmission and testes damage. In the case of GS-5700, two doses of 25 µg DNA by the intramuscular route given two weeks apart protected mice from challenge with wild-type ZIKV, including no detectable vRNA in testes while one dose of 10,000 ffu 10-del ZIKV also resulted in no detectable vRNA in testes post ZIKV challenge. However, in a pregnancy model, vaccination with either NS1 ZIKV or mRNA LNP followed by wild-type ZIKV challenge, resulted in a minority of fetuses positive for Zika vRNA, but no infectious ZIKV was detected. Again, interpretation of these results is difficult as the significance of detection of ZIKV RNA is not fully-understood. While the studies undertaken are elegant at the research level, the mouse reproductive system is not a good model for humans and so interpretation must be done with care. Nonetheless, in the pregnancy model, it was significant that there was an inverse relationship between neutralization titer and virus found in the placenta suggesting that an appropriately high neutralization titer in serum will protect against infection of reproductive tissues.

## Therapeutic vaccination

While much progress has been made, there is still a long way to go to get a prophylactic vaccine to licensure. An alternative approach is therapeutic vaccination. A number of studies have generated and characterized mouse and human monoclonal antibodies (mabs) against ZIKV. The range of epitopes of ZIKV appears very similar to that of other flaviviruses, with similar binding affinities, although for most antibodies require higher concentrations of antibodies to elicit neutralization activity as compared to other flaviviruses. This may explain, in part, why higher titers of ZIKV neutralizing antibodies are needed for protection in the mouse model (around 100) as compared to the low titers (about 10) needed for the three licensed flavivirus vaccines; care is needed in interpretation of these data as there is no neutralization assay calibrated to an international standard for ZIKV as there is for the licensed flavivirus vaccines. Nonetheless, both mouse^19^ and human^20^ mab studies in mice and NHPs, respectively, suggest that therapeutic vaccination has potential. In particular, Sapparapu et al.^19^ prevented maternal-fetal transmission in the mouse model, while Magnani et al.^20^ used a cocktail of three neutralizing mabs (two recognize domain III and one recognizes domain II) given one day before ZIKV challenge resulted in no detectable ZIKV viremia in ZIKV challenged NHPs. Thus, passive administration of human mabs provide a potential important approach to short-term protective immunity, potentially six months with current antibody engineering technology, that may be particularly useful for pregnant women or rapid onset protection while waiting for protective immune responses from active immunization.

Overall, great progress has been made with development of a Zika vaccine. However, a number of issues remain to be resolved. As indicated above, neutralization is the surrogate for protective immunity for the licensed flavivirus vaccines and current evidence suggests that neutralizing antibodies are likely to be the surrogate for protection for ZIKV vaccines too, however, this remains to be confirmed in clinical trials. Furthermore, evidence to date also suggests that higher levels of neutralizing antibodies are required to protect animals (and humans?) against ZIKV infection compared to other flaviviruses. Nonetheless, it should be remembered that the vaccine developers are using different neutralization assays, which makes any comparisons between candidate Zika vaccines and/or Zika with other flaviviruses very difficult. In addition, significant progress has been made in developing standards for vaccine testing, including a nucleic acid standard developed by the Paul Erhlich Institute, which will enable viral RNA data to be quantified based on international units, and international antibody standards and a proficiency panel for neutralization assays are in development by the National Institute for Biological Standards and Control. If neutralizing antibodies are confirmed as the surrogate of protection, these antibodies should enable a neutralization titer in international units to be calculated for a surrogate for protection. Finally, as Zika vaccine development moves forward, it appears that candidate vaccines (although possibly not a live vaccine) require multiple doses to give high titers of neutralizing antibodies. One possible approach in the future may be heterologous prime-boost where one vaccine platform is used for the first dose and a different vaccine platform is used for the second dose, which may improve induction of a long term protective immune response.

## References

[1] Barrett ADT (2016). Zika vaccine candidates progress through nonclinical development and enter clinical trials. NPJ Vaccines.

[2] World Health Organization. http://www.who.int/immunization/research/development/WHO_UNICEF_Zikavac_TPP_Feb2017.pdf?ua=1.

[3] Tebas, P. et al. Safety and immunogenicity of an anti-Zika virus DNA vaccine—preliminary report. *N. Engl. J. Med*. 10.1056/NEJMoa1708120 (2017). 10.1056/NEJMoa1708120 PMC6824915 34525286

[4] Modjarrad, K.et al. Preliminary aggregate safety and immunogenicity results from three trials of a purified inactivated Zika virus vaccine candidate: phase 1, randomised, double-blind, placebo-controlled clinical trials. *Lancet*. 10.1016/S0140-6736(17)33106-9 (2017). 10.1016/S0140-6736(17)33106-9 PMC5884730 29217375

[5] Gaudinski, M. R. et al. Safety, tolerability, and immunogenicity of two Zika virus DNA vaccine candidates in healthy adults: randomised, open-label, phase 1 clinical trials. *Lancet*. 10.1016/S0140-6736(17)33105-7 (2017). 10.1016/S0140-6736(17)33105-7 PMC6379903 29217376

[6] Muthumani, K. et al. In vivo protection against ZIKV infection and pathogenesis through passive antibody. *npj Vaccines*. 10.1038/NPJVACCINES.2016.21 (2016). 10.1038/npjvaccines.2016.21 PMC5707885 29263859

[7] Larocca RA (2016). Vaccine protection against Zika virus from Brazil. Nature.

[8] Abbink P (2016). Protective efficacy of multiple vaccine platforms against Zika virus challenge in rhesus monkeys. Science.

[9] Dowd KA (2016). Rapid development of a DNA vaccine for Zika virus. Science.

[10] Marston HD, Lurie N, Borio LL, Fauci AS (2016). Considerations for developing a Zika virus vaccine. N. Engl. J. Med..

[11] Thomas SJ, L’Azou M, Barrett AD, Jackson NA (2016). Fast-track Zika vaccine development—is it possible?. N. Engl. J. Med..

[12] Chan C (2017). A live-attenuated Zika virus vaccine candidate induces sterilizing immunity in mouse models. Nat. Med..

[13] Shan C (2017). A single-dose live-attenuated vaccine prevents Zika virus pregnancy transmission and testis damage. Nat. Commun..

[14] Richner JM (2016). Modified mRNA vaccines protect against Zika virus infection. Cell.

[15] Pardi N (2017). Zika virus protection by a single low-dose nucleoside-modified mRNA vaccination. Nature.

[16] Abbink P (2017). Durability and correlates of vaccine protection against Zika virus in rhesus monkeys. Sci. Transl. Med..

[17] Griffin BD (2017). DNA vaccination protects mice against Zika virus-induced damage to the testes. Nat. Commun..

[18] Richner JM (2017). Vaccine mediated protection against Zika virus-induced congenital disease. Cell.

[19] Sapparapu G (2016). Neutralizing human antibodies prevent Zika virus replication and fetal disease in mice. Nature.

[20] Magnani DM (2017). Neutralizing human monoclonal antibodies prevent Zika virus infection in macaques. Sci. Transl. Med..

